# Medical Colleges—Opening of the Annual Sessions

**Published:** 1888-10

**Authors:** 


### *MEDICAL COLLEGES—OPENING OF THE ANNUAL SESSIONS.*

This is the season of the year in which the medical colleges throw open their doors for the admission of new aspirants for medical honors. The medical colleges in this city inaugurated their autumn and winter course under the usually flattering auspices.

The opening of the session of the Medical Department of Niagara University took place on Wednesday evening, September 19th, at the amphitheater in the College Building, on Ellicott street. The capacity of the building was fully tested, as is usual on this occasion, and the exercises were highly interesting. The opening address was given by Professor Henry D. Ingraham, on

"Progress in Medicine," and was fully worthy of the ability of the writer.

The trustees availed themselves of the opportunity to confer a merited honor on the distinguished President of the Medical Faculty, Dr. Cronyn. The Rev. M. J. Kircher, Secretary of the Trustees, in an appropriate address given in Latin, presented Dr. Cronyn to the Chancellor, the Rt. Rev. Bishop Ryan, who conferred on him the degree of Doctor in Philosophy, in recognition of the distinguished services he has rendered to the profession and the community.

This feature of the exercises were novel, and the new *philosopher* acknowledged the compliment in a few well-chosen remarks, in which assurances were given of renewed devotion to duty in the remaining years of his life. We beg to tender our congratulations to President Cronyn, and to assure him that the high honor thus conferred is not only fully merited and also appreciated by the faculty of which he is the honored head, but by the profession.

The opening of the annual session of the Buffalo Medical College took place on Monday, September 24th, Dr. John H. Pryor giving the address.

